# Isolated Neutropenia Due to Immune Checkpoint Inhibitors

**DOI:** 10.7759/cureus.45674

**Published:** 2023-09-21

**Authors:** Ahmad Jalil, Javariya Zaffar, Aimal Waqas, Shayan Butt

**Affiliations:** 1 Internal Medicine, King Edward Medical University, Lahore, PAK; 2 Internal Medicine, Rehman Medical College, Peshawar, PAK; 3 Internal Medicine, Foundation University Medical College, Islamabad, PAK; 4 Internal Medicine, Mayo Clinic, Jacksonville, USA

**Keywords:** immune related adverse event (irae), immunotherapy-related adverse events, chemotherapy-induced neutropenia, immune checkpoint inhibitors (icis), immune-checkpoint inhibitors

## Abstract

In this article, we explore the correlation between immune checkpoint inhibitors (ICIs) and neutropenia. Immune checkpoint inhibitors have revolutionized cancer treatment and management by maximizing the innate abilities of the immune system. However, this therapeutic potential is accompanied by a range of immune-related adverse effects (irAEs), including neutropenia, which is a rare but potentially life-threatening side effect of this mode of cancer treatment. Through an in-depth analysis of various case reports, we have compiled a detailed table summarizing the occurrences of neutropenia associated with different ICIs, the grades of neutropenia, treatments used, and patient outcomes. Management of neutropenia must include an approach based on early diagnosis of the condition and a treatment based on its severity. This review discusses different therapeutic interventions, ranging from the administration of corticosteroids and intravenous immunoglobulin (IVIG) to the use of granulocyte colony-stimulating factor (filgrastim) and, in very severe cases, a stem cell transplant. We have also enlisted salient side effects caused by these interventions. Our findings emphasize that while neutropenia is a relatively rare adverse effect of ICIs, its severity necessitates increased awareness among healthcare professionals. As ICIs continue to be seen as an integral component of cancer therapy, a comprehensive understanding of neutropenia as a side effect and its management is critical for optimizing patient outcomes. A crucial purpose of this review is to highlight the need to achieve a balance between acquiring the therapeutic benefits of various treatment strategies for irAEs and considering their potential side effects, especially with the use of steroids. Achieving this equilibrium is very important in optimizing patient care during immunotherapy, as these irAE management options can both mitigate the neutropenia triggered by ICIs and potentially give rise to secondary complications. Therefore, a careful assessment of the risks and benefits associated with each treatment approach is essential in tailoring irAE management.

## Introduction and background

Immune checkpoint inhibitors (ICIs) are a class of regulatory drugs that are designed to block specific proteins on the surface of immune cells or cancer cells, thereby enhancing the immune system's ability to recognize and destroy cancer cells. Two of the most well-known immune checkpoint proteins targeted by ICIs are programmed cell death protein 1 (PD-1) and cytotoxic T-lymphocyte-associated protein 4 (CTLA-4). ICIs targeting these two proteins are involved in inhibiting T-cell activation and, therefore, attack the malignant cells. In the realm of medicine, ICIs have demonstrated significant clinical benefits across a wide range of malignancies, establishing them as one of the most gratifying breakthroughs in this field. However, immunotherapies operate through modulating the immune system, which can adversely trigger immune-related adverse events (irAEs) [1]. These irAEs can manifest as dermatologic, gastrointestinal, hepatic, and pulmonary reactions. Hematologic immune-related adverse events (heme-irAEs), in particular, have been progressively reported in the literature with a reported fatality rate of 12%. According to the French pharmacovigilance databases, neutropenia, autoimmune hemolytic anemia, and immune thrombocytopenia were the most commonly occurring heme-irAEs [2].

Recent research has revealed isolated neutropenia to be the rarest but the most deleterious manifestation resulting from the use of ICI [3]. According to the National Cancer Institute Common Terminology Criteria for Adverse Events, chemotherapy-induced neutropenia (CIN) can be divided into four grades: Grade 1 with an absolute neutrophil count (ANC) of 1500-2000 cells/mm^3^, Grade 2 with an ANC of 1000-1500 cells/mm^3^, Grade 3 with an ANC of 500-1000 cells/mm^3^, Grade 4 with an ANC <500 cells/mm^3^ [4]. Isolated neutropenia secondary to ICI use presents a reasonable concern to patients’ safety given the limited amount of literature related to it. It is important to note that the incidence and severity of these heme-irAEs can vary among individuals and depend on factors such as the type of cancer being treated, the specific ICI used and the overall health of the patient [5]. In this review, we have compiled a summary of 20 published cases of ICI-induced neutropenia for the treatment of various cancers, encompassing lung cancer, melanoma, multiple myeloma, bladder cancer, and Hodgkin lymphoma. Furthermore, we have explored several aspects of irNeutropenia, which include the number of cycles before onset of neutropenia, the infectious complications that may arise from low neutrophil levels as well as effective strategies for managing this condition. Despite terminating the immune checkpoint blockade, neutropenia persisted in most of the cases, which calls for careful consideration and management.

## Review

According to this review of 20 published cases (Table 1), ICI-induced neutropenia had an equal occurrence rate in males and females. The youngest individual reported to experience neutropenia was 32 years old, while the oldest patient was 82 years old. In terms of underlying oncological conditions, lung cancer accounted for the highest number of cases treated with immunotherapy followed by melanoma and bladder cancer. A singular instance of Hodgkin’s lymphoma was also documented. The most frequently utilized ICIs were pembrolizumab and nivolumab. Nivolumab was commonly prescribed for cases involving lung cancer, melanoma, and even Hodgkin’s lymphoma. Pembrolizumab was initiated for a variety of cancers including carcinoma of the lungs, multiple myeloma, and bladder cancer. Ipilimumab, primarily, was administered for the treatment of melanoma, and durvalumab showed effectiveness in treating squamous cell lung carcinoma. Development of abnormal LFTs was seen in a few patients on nivolumab for lung cancer.

**Table 1:** Summary of published cases with isolated neutropenia due to immune checkpoint inhibitor therapy

Age/gender	Immune checkpoint inhibitor used	Type of malignancy	Onset of neutropenia (number of cycles before neutropenia)	Neutropenia grade/ANC	Management of neutropenia	ICI management	Outcome/complications of neutropenia	Additional comments	Reference
73/F	Pembrolizumab	Pulmonary adenocarcinoma, stage 4, demonstrating 90% PD-L1 positivity	2 cycles	Grade 4, ANC 0.0	Granulocyte colony-stimulating factor (G-CSF) (stopped due to inefficacy). Intravenous solumedrol 40mg q6H. Immunoglobulins. Cyclosporine A (held after 3 days because of febrile neutropenia)	Stopped	ANC began recovering 6.5 weeks after the initial infusion of pembrolizumab. Neutropenia resolved after another dose of G-CSF. Steroids tapered off	Comorbidities: myositis, Crohn’s disease, and hypothyroidism	Barbacki et al. [6]
64/F	Pembrolizumab	Urothelial tumor pT1 grade III	1 cycle	Before starting immunotherapy neutrophil count: 9600/mm^3^. After 1^st^ cycle and 3 days before the 2^nd^: 1400/mm^3^. During subsequent cycles: between 500/mm^3^ and 2200/mm^3^	None	Continued	Patient’s latest ANC was reported to be 660/mm^3^	Lung nodules, seen 12 weeks after initiation of Pembrolizumab, were not relieved with antibiotics; no infectious cause was identified, and nodule biopsy showed hypersensitivity pneumopathy due to immunotherapy. Good response with steroids and cotrimoxazole	Laurain et al. [7]
49/F	Pembrolizumab	Multiple myeloma; R-ISS stage II, kappa light chain with 24% marrow plasma cells	2 cycles	Grade 4, ANC 0	Neutropenia did not respond to G-CSF, IVIG, prednisone 1–2mg/kg, methylprednisolone 1–2mg/kg), rituximab, cyclosporine, tocilizumab; autologous hematopoietic stem cell transplant (ASCT) performed after persistent neutropenia for 64 days	Stopped	The patient sustained many infections while she was neutropenic, including sinusitis, blepharitis and periorbital cellulitis, HSV oral ulceration, suspected H. pylori gastritis, and rapidly progressive necrotizing pseudomonal facial cellulitis. Neutrophil engraftment was established 9 days after ASCT		Bryant et al. [8]
73/M	Nivolumab	Lung adenocarcinoma	5 cycles	The steady decline in neutrophil count since initiating nivolumab. ANC 480/mm^3^ (Grade 4) prior to 6^th^ cycle and treatment suspended. ANC 0/mm^3^ 2 days later	Antibiotics, G-CSF, methylprednisolone (1mg/kg)	Stopped	Hospitalization for neutropenic fever and diarrhea. The fever subsided after 24 hours; neutropenia persisted for a week before improvement	The patient also experienced Crohn's and COPD exacerbation along with the development of Grade 4 neutropenia. He experienced arrhythmias and unfortunately expired 13 days after admission	Turgeman et al. [9]
74/F	Nivolumab	Lung adenocarcinoma	2 cycles	Mild neutropenia (1400/mm^3^) on the day of her 2^nd^ dose. Severe neutropenia later on	G-CSF, IVIG, and oral and IV steroids. Prednisone (1.5mg/kg); later switched to IV methylprednisolone (initially at 2mg/kg, later at 3mg/kg)	Stopped	Hospitalization with ecthyma gangrenosum, received IV antibiotics. The neutrophil count began to recover at higher doses of IV methylprednisone (3mg/kg daily) on day 14 of hospitalization, after remaining refractory at previous treatments, including a lower dose of IV methylprednisone (1.5 mg/kg daily and 2mg/kg daily)	Elevated liver function tests (LFTs) along with neutropenia	Tabchi et al. [10]
59/M	Ipilimumab and nivolumab	Melanoma stage IV	2 cycles	Grade 4	G-CSF, IVIG (1g/kg), IV, methylprednisolone (2mg/kg), mycophenolate mofetil	Stopped	ANC normalized with mycophenolate, transitioned to oral glucocorticoids and continued to receive mycophenolate mofetil to allow for a steroid taper	Also developed grade 3 hepatitis and colitis. According to the case report, this was the first incident where a second-line immune modulator, i.e., mycophenolate was used to treat ICI-induced neutropenia	Meti et al. [11]
60/F	Durvalumab	Lung cancer (squamous cell, cT3N2M0)	16 cycles	Grade 4	G-CSF, prednisone, antibiotics (amoxicillin/clavulanic acid and clarithromycin)	Stopped	Steroids were slowly tapered off and patient’s neutrophil count remained normal at the 2-year follow-up		Bandura et. Al. [12]
78/M	Pembrolizumab	Lung cancer (squamous) with brain metastasis	2 cycles	Grade 4	G-CSF, steroids	Stopped	No improvement after 14 days of G-CSF. IV methylprednisone was added in gradually increasing doses, neutrophil count returned to normal after 10 days	Positive sputum culture for Staphylococcus haemolyticus. Fungal GM test was also positive. Antibiotics and antifungals were added	Tan et al. [13]
72/M	Pembrolizumab	Non-small cell lung cancer	4 cycles	Grade 4	G-CSF, steroids	Stopped	Late neutropenia 118 days after final administration of pembrolizumab, not improved on G-CSF, resolution with methylprednisolone (2000 mg/day)	Developed ICI-induced hypothyroidism, pneumonitis and hepatitis before developing neutropenia	Nakako et. l. [14]
57/M	Nivolumab	Non-small cell lung cancer, metastasized	2 cycles	Grade 3	G-CSF	Stopped	Initial asymptomatic grade 3 neutropenia converted into febrile grade 3 neutropenia on day 32 after the first dose of nivolumab, improved with stopping ICI and concurrent treatment with G-CSF. However, on day 77, the patient had to be retreated with G-CSF as neutrophil count became 500/mL, which normalized immediately after the treatment	Grade 2 liver dysfunction seen, responded well to glucocorticoids (initially prednisolone 0.5 mg/kg/day, later methylprednisolone 2mg/kg as LFTs worsened)	Hisamatsu et al. [15]
42/F	Ipilimumab	Metastatic melanoma	4 cycles	Grade 4, ANC 38/mL	G-CSF antibiotics (e.g., vancomycin, cefepime, levofloxacin), antifungals (e.g., fluconazole) prednisone (1mg/kg). Change of corticosteroid to dexamethasone (8mg IV every 6 hours) on second hospitalization. Cyclosporine 100mg and IVIG 1g/kg	Stopped	3 courses of IVIG and multiple infusions of G-CSF before pancytopenia resolved. Neutrophil count became normal 2 weeks after the last treatment with G-CSF and 4 weeks after the last IVIG course	Patient developed pancytopenia due to ICI	Akhtari et al. [16]
59/F	Pembrolizumab	Metastatic bladder cancer (high-grade urothelial cancer with invasion of muscularis propria)	19 cycles	Grade 4, ANC 10/mL	Zosyn, fluconazole, G-CSF (300mg daily), IV methylprednisolone (2mg/kg/day)	Stopped	Neutrophil count recovered after 13 days. Antibiotics were stopped. Methylprednisolone switched to oral prednisolone (150 mg daily) and was tapered off over next 2 months	Developed grade 3 mucositis and hypotension requiring intense fluid therapy	Baleiras et al. [17]
32/F	Nivolumab	Hodgkin’s lymphoma (Stage IV, classic nodular)	15 months therapy (cycles not reported)	Grade 1, ANC 1500	IVIG (500 mg/m^2^ for 2 days) and dexamethasone (40 mg/day for 4 days)	Nivolumab was held and restarted multiple times during the treatment, depending on the cell counts	Neutrophils and platelet count were recovered after withholding treatment	Concurrent thrombocytopenia with possible idiopathic thrombocytopenic purpura (ITP) and possibly autoimmune neutropenia	Bulbul et al. [18]
75/F	Nivolumab	Melanoma (Stage IIIB)	14 cycles	Gradually worsening neutropenia reaching Grade 4 with ANC 0/mL	Initially prednisolone, later methylprednisolone. IVIG, G-CSF, mycophenolate. Vancomycin, cefepime	Stopped	High dose steroid therapy- prednisone initiated at 60 mg and later increased to 80 mg. Steroid taper could not be completed due to worsening neuropathy. Switched to methylprednisolone 60 mg daily, which resolved neuropathy but worsened myopathy. Neutropenia persisted despite high dose corticosteroids, immunosuppression, and IVIG. Comfort measures opted per goals of care. Patient passed away 48 days after first episode of neutropenia	Developed transaminitis and neuropathy along with neutropenia from Nivolumab use. Also developed proximal muscle weakness from steroid use	Patel et al. [19]
35/M	Ipilimumab	Melanoma	3 cycles	Grade 4, ANC 0/mL	Methylprednisolone. G-CSF	Not reported	Neutrophil count recovered after 16 days		Wozniak et al. [20]
74/M	Pembrolizumab	Metastatic non-small cell lung cancer	4 cycles	Grade 4, ANC 0/mL	Antibiotics, 1m/kg steroids, G-CSF	Stopped	Neutropenia count recovered initially with the given regimen. However, it relapsed after 8 weeks of recovery	CRP and inflammatory cytokines demonstrated a rise during the neutropenic period	Naqash et al. [21]
54/M	Ipilimumab	Stage IIIb melanoma	4 cycles	Grade 4, ANC 0/mL	Initially prednisone (60mg twice a day), cyclosporine (125mg twice daily), IVIG (40mg daily) G-CSF (5mcg/kg daily). Later rabbit anti-thymocyte globulin (ATG) (15mg/kg twice daily), cyclosporine (2.5mg/kg twice daily), prednisone, G-CSF	Stopped	Neutropenia persisted with the initial treatment due to which the patient was switched to a regimen on ATG/cyclosporine/prednisone. ANC improved to 0.1/mL. G-CSF was added, and the ANC gradually improved to 5.2/mL. Prednisone was tapered down to 5mg by the 19 week which led to neutropenia relapse. Prednisone’s dose was increased, and slower taper was tried by which ANC stabilized	Developed maculopapular rash within 1^st^ week of ipilimumab infusion, which was improved by 2.5% hydrocortisone cream. Patient also experienced decreased libido, treated with 5g topical testosterone	Ban-Hoefen et al. [22]
82/M	Pembrolizumab	Pleomorphic lung cancer	3 cycles	Grade 4, ANC 68/mL	Initially meropenem, vancomycin, micafungin. Later G-CSF (75 μg/body for 4 days)	Stopped	Patient hospitalized for febrile neutropenia, 22 days after last pembrolizumab cycle and started on initial treatment. Anti-neutrophil antibody detected in peripheral blood. G-CSF administered on the 5th hospital day. Neutrophil count improved on 7^th^ day of treatment. G-CSF discontinued. Patient discharged after 15 days	Organizing pneumonia showing intraluminal polypoid fibrosis in alveoli was seen on transbronchial lung tissue biopsy from the right middle lobe which did not improve with antibacterial medication. Patient developed diplopia on 7^th^ day of discharged, which was diagnosed as ocular myasthenia gravis secondary to pembrolizumab treatment and was started on pyridostigmine 60mg three times a day	Tozuka et al. [23]
63/M	Nivolumab	Lung squamous carcinoma and right pleural metastasis	Not mentioned	Grade 4, ANC 0.43/mL	Methylprednisolone, G-CSF, tigecycline meropenem	Stopped	The patient developed grade 4 neutropenia, 3 weeks after the last nivolumab treatment. Resolved by G-CSF. Regression of carcinoma by methylprednisolone use	Patient also developed fever and purulent sputum. Microbiological culture assay revealed multidrug-resistant Klebsiella pneumoniae infection. Chest CT revealed metastasis to lower left lung as well, which was controlled in <2 weeks by tigecycline and meropenem use	Liu et al. [24]

The most popular treatment modalities include steroids, IVIG, GM-CSF, and immunosuppressants like cyclosporine, mycophenolate, etc. The choice of therapies and their combinations can vary widely based on the individual patient's condition, the underlying cause, and the severity of the neutropenia. Multidisciplinary collaboration between different physicians including hematologists, infectious disease specialists, and other healthcare providers is often necessary to tailor treatment plans to each patient's unique needs.

Steroids have the most important role in mitigating neutropenia, caused by ICI therapy, based on current published literature. Needless to say, stopping the offending ICI with the onset of neutropenia is imperative. Steroids can present challenges due to their side effects when used for a long period of time and thus, balancing with their desired effects can be tricky. A case study by Patel et al. [19] exemplifies this, where the administration of steroids led to severe myopathy in a 75-year-old female who was taking nivolumab for Stage IIIB melanoma. This case is particularly interesting as the patient initially developed severe neuropathy while taking immunotherapy. Later, the introduction of steroid therapy resulted in marked improvement in the neuropathy but the use of steroids also caused severe myopathy, complicating the patient's clinical course. Attempting to taper the steroid dosage would exacerbate the neuropathy. Thus, it proved difficult to decide between whether to keep her on steroids or taper them off. However, despite the incorporation of steroids, intravenous immunoglobulin (IVIG), and other immunosuppressive agents into the treatment regimen, the patient’s neutropenia persisted. Ultimately, comfort care was initiated and this was reported as a mortality. This case serves as a reminder of the difficulty faced in managing neutropenia and its associated complications, where the use of therapeutic agents can introduce significant side effects. It brings into focus the critical need for personalized treatment strategies and the consideration of individual patient factors when deciding on a management plan.

As noted above, the initial treatment for immune-mediated neutropenia is discontinuing the suspected ICI, except for one case presented by Laurain et al. [7], where ICI therapy was continued despite neutropenia. An interesting aspect of this case was that the patient developed pulmonary nodules as a side effect of immunotherapy as confirmed on biopsy, which revealed hypersensitivity pneumonitis due to immunotherapy. The patient showed improvement on steroids and co-trimoxazole.

In certain severe instances where standard therapies like steroids, IVIG, and G-CSF do not yield positive results in addressing neutropenia, the option of immunosuppressive agents is available. Cyclosporine [6,8,22], mycophenolate [11,19], and tocilizumab [8] have been used to combat irNeutropenia (immune-related neutropenia). In all of these reports, the patients already were on steroids. Immunosuppressive agents were not used as monotherapy in any of the cases. Bryant et al. [8] showed that with the use of immunosuppressants, patients had infectious complications, e.g., pseudomonal facial cellulitis with the use of cyclosporine [8].

In rare instances, a stem cell transplant may be considered. One such case, as detailed by Bryant et al. [8] involves the application of pembrolizumab to treat multiple myeloma. In this scenario, a 49-year-old female patient encountered Grade 4 neutropenia after undergoing 2 cycles of pembrolizumab treatment. As a result, the administration of pembrolizumab was stopped, and the patient's initial treatment plan included the use of steroids, IVIG, and G-CSF, along with rituximab, cyclosporine, and tocilizumab. Throughout the treatment duration, the patient experienced several infections, including a very aggressive case of pseudomonal facial cellulitis. Eventually, due to the persistence of neutropenia for over two months, the patient received an autologous hematopoietic stem cell transplant after which the neutropenia resolved.

Filgrastim, a form of G-CSF, was a common intervention across all cases, but its effectiveness was limited in almost all cases. Bulbul et al. [18] did not utilize GM-CSF and resorted only to IVIG and dexamethasone, resulting in a favorable response. Clearly, in almost all of the other cases, GM-CSF was utilized and two published cases reported no significant response when GM-CSF was used as monotherapy [6,8]. GM-CSF, which plays a crucial role in the production and activation of white blood cells, holds promise in various clinical contexts, but its effectiveness as a single treatment modality may not be enough to cause considerable improvement for immunotherapy-related neutropenia.

In cases where patients developed febrile neutropenia, a condition characterized by a fever accompanying a low absolute neutrophil count, antimicrobial therapy was prescribed as a low neutrophil count left the patients vulnerable to infections. The initial step in managing febrile neutropenia involved the administration of broad-spectrum antibiotics. The choice of a broad-spectrum antibiotic is based on the need to quickly address the infection while waiting for more specific information about the causative agent. Once the patient's condition stabilized and laboratory tests yielded results, efforts were made to isolate the infectious agent responsible for the fever and neutropenia. Identifying the specific microorganism causing the infection is essential for tailoring the treatment regimen to be as targeted and effective as possible. However, it is important to note here that a study performed by Derosa et al. [25] showed that the application of antibiotics can disturb the distribution of gut microbes, leading to the development of resistance to ICIs, especially anti-PD-1 inhibitors. Another study suggested that antibiotic therapy used before starting patients on ICI is associated with worse rates of response to immunotherapy and survival. An antibiotic therapy taken concurrently with ICI therapy appeared to be safe [26]. Therefore, care must be taken while prescribing antibiotics especially if they are being prescribed prior to ICI treatment.

## Conclusions

Extensive research endeavors are crucial to understanding the complete spectrum of immune-related adverse events and identifying the most effective strategies for managing these occurrences, especially when dealing with the rarest manifestations, one of which is isolated neutropenia, and assessing the overall risk/benefit profile of utilizing ICIs in cancer patients. Heightened awareness regarding neutropenia can lead to earlier detection, improved management, and ultimately better outcomes for patients. Since neutropenia is a multifaceted phenomenon, addressing it demands a collaborative effort among specialists from diverse fields to work together as a cohesive team to revolutionize the understanding of these complexities. The goal is to optimize the use of ICIs, minimize toxicity, and maximize their beneficial effects.

With the available limited literature, generally, stopping the offending immunotherapy and steroids with or without IVIG seems to be the mainstay and commonly employed first step in the management of isolated neutropenia. The preferred steroids are either methylprednisolone at a dose of at least 1-2 mg/kg or prednisone at a dose of at least 1-2 mg/kg. IVIG is of particular interest and monotherapy has not been tried, but still, we conclude that in severe neutropenia, IVIG should not be tried as a stand-alone therapy. A general reflex is to give GM-CSF; however, interestingly, GM-CSF did not show promising effects in all of the cases. The same could be said about immunosuppressants in general. Where these drugs are beneficial to have in the arsenal, monotherapy has never been tried and complications have frequently been reported.

We also conclude that neutropenia may last several days to a week before any response can be substantiated. This can lead to prolonged hospitalization and/or frequent visits to outpatient clinics which can result in caregiver fatigue on top of psychological dismay of the patient. When dealing with complications of neutropenia, patients need to be hospitalized and management should be done under isolation especially when infective organisms are involved. Our review aims to highlight the importance of early recognition of neutropenia among patients on ICI treatment through a review of published cases on this side effect. Stopping the ICI treatment is the most effective initial step followed by other therapies like corticosteroids, IVIG, G-CSF, and immunosuppressants; depending upon the grade of neutropenia. Mostly, corticosteroids are able to treat neutropenia based on our review. In patients who develop secondary infections, antimicrobials may be prescribed according to the nature of the infection. When all of the other therapies fail to produce recovery, an autologous stem cell transplant (ASCT) could become necessary. A multidisciplinary approach is always warranted and a hematologist should always be consulted. 

A crucial aspect of this exploration is to understand the relationship between autoimmune conditions and the administration of ICIs. It is essential to discern whether irAEs from ICIs trigger autoimmunity or if preexisting autoimmune conditions may exacerbate after initiating ICI therapy. Understanding this association holds profound significance for tailoring cancer treatments and influencing patient outcomes in a positive and impactful manner.

## References

[1] Ramos-Casals M, Brahmer JR, Callahan MK (2020). Immune-related adverse events of checkpoint inhibitors. Nat Rev Dis Primers.

[2] Delanoy N, Michot J-M, Comont T (2019). Haematological immune-related adverse events induced by anti-PD-1 or anti-PD-L1 immunotherapy: a descriptive observational study. Lancet Haematol.

[3] Ghanem P, Marrone K, Shanbhag S, Brahmer JR, Naik RP (2022). Current challenges of hematologic complications due to immune checkpoint blockade: a comprehensive review. Ann Hematol.

[4] Ba Y, Shi Y, Jiang W (2020). Current management of chemotherapy-induced neutropenia in adults: key points and new challenges: Committee of Neoplastic Supportive-Care (CONS), China Anti-Cancer Association Committee of Clinical Chemotherapy, China Anti-Cancer Association. Cancer Biol Med.

[5] Kanji S, Morin S, Agtarap K (2022). Adverse Events Associated with Immune Checkpoint Inhibitors: Overview of Systematic Reviews. Drugs.

[6] Barbacki A, Maliha PG, Hudson M, Small D (2018). A case of severe Pembrolizumab-induced neutropenia. Anticancer Drugs.

[7] Laurain PA, Landrin T, Souidi S, Beuzeboc P, Scotté F (2020). Atypical extended immune-related neutropenia in patient treated with pembrolizumab. Eur J Cancer.

[8] Bryant AR, Perales MA, Tamari R, Peled JU, Giralt S (2018). Severe pembrolizumab-associated neutropenia after CD34(+) selected allogeneic hematopoietic-cell transplantation for multiple myeloma. Bone Marrow Transplant.

[9] Turgeman I, Wollner M, Hassoun G, Bonstein L, Bar-Sela G (2017). Severe complicated neutropenia in two patients with metastatic non-small-cell lung cancer treated with nivolumab. Anticancer Drugs.

[10] Tabchi S, Weng X, Blais N (2016). Severe agranulocytosis in a patient with metastatic non-small-cell lung cancer treated with nivolumab. Lung Cancer.

[11] Meti N, Petrogiannis-Haliotis T, Esfahani K (2018). Refractory Neutropenia Secondary to Dual Immune Checkpoint Inhibitors That Required Second-Line Immunosuppression. J Oncol Pract.

[12] Bandura A, Kurlapski M, Kamińska M, Dziadziuszko R, Jassem J (2022). Profound neutropenia related to consolidation durvalumab treatment following concurrent radiochemotherapy in a patient with stage III non-small‑cell lung cancer. Pol Arch Intern Med.

[13] Tan Q, Liu L, Huang Y, Dong X, Chen L (2022). Case report: A rare case of neutropenia caused by pembrolizumab in squamous lung cancer and literature review. Front Oncol.

[14] Nakako S, Nakashima Y, Okamura H (2022). Delayed immune-related neutropenia with hepatitis by pembrolizumab. Immunotherapy.

[15] Hisamatsu Y, Morinaga R, Watanabe E, Ohtani S, Shirao K (2020). Febrile Neutropenia in a Patient with Non-Small Cell Lung Cancer Treated with the Immune-Checkpoint Inhibitor Nivolumab. Am J Case Rep.

[16] Akhtari M, Waller EK, Jaye DL, Lawson DH, Ibrahim R, Papadopoulos NE, Arellano ML (2009). Neutropenia in a patient treated with ipilimumab (anti-CTLA-4 antibody). J Immunother.

[17] Miranda Baleiras M, Vasques C, Pinto M, Miranda H, Martins A (2022). Pembrolizumab-Induced Autoimmune Grade 4 Neutropenia in a Patient With Advanced Bladder Cancer: A Case Report. Cureus.

[18] Bulbul A, Mustafa A, Chouial S, Rashad S (2017). Idiopathic thrombocytopenic purpura and autoimmune neutropenia induced by prolonged use of nivolumab in Hodgkin's lymphoma. Ann Oncol.

[19] Patel R, Pai L (2020). Immunotherapy-induced severe neutropenia with neurotoxicity: A case of a 75-year-old woman with ulcerative colitis diagnosed with melanoma. J Oncol Pharm Pract.

[20] Woźniak S, Mackiewicz-Wysocka M, Krokowicz Ł, Kwinta Ł, Mackiewicz J (2015). Febrile neutropenia in a metastatic melanoma patient treated with ipilimumab - case report. Oncol Res Treat.

[21] Naqash AR, Appah E, Yang LV (2019). Isolated neutropenia as a rare but serious adverse event secondary to immune checkpoint inhibition. J Immunother Cancer.

[22] Ban-Hoefen M, Burack R, Sievert L, Sahasrabudhe D (2016). Ipilimumab-Induced Neutropenia in Melanoma. J Investig Med High Impact Case Rep.

[23] Tozuka T, Sugano T, Noro R (2018). Pembrolizumab-induced agranulocytosis in a pulmonary pleomorphic carcinoma patient who developed interstitial lung disease and ocular myasthenia gravis. Oxf Med Case Reports.

[24] Liu C, Ding L, Zhu YH, Chen C (2017). A rare case of lung carcinoma acquires multidrug-resistant Klebsiella pneumoniae pneumonia radiologically mimicking metastasis caused by nivolumab therapy-associated neutropenia. Ther Clin Risk Manag.

[25] Derosa L, Hellmann MD, Spaziano M (2018). Negative association of antibiotics on clinical activity of immune checkpoint inhibitors in patients with advanced renal cell and non-small-cell lung cancer. Ann Oncol.

[26] Pinato DJ, Howlett S, Ottaviani D (2019). Association of Prior Antibiotic Treatment With Survival and Response to Immune Checkpoint Inhibitor Therapy in Patients With Cancer. JAMA Oncol.

